# Machine Learning–Based Diagnosis of Cryptococcal Meningitis and Tuberculous Meningitis: A Single-Center Retrospective Clinical Study

**DOI:** 10.1093/ofid/ofaf217

**Published:** 2025-04-08

**Authors:** Fangbo Lin, Cheng Chen, Yuan Li, Hua Liu

**Affiliations:** Rehabilitation Medicine Department, The Affiliated Changsha Hospital of Xiangya School of Medicine, Central South University (The First Hospital of Changsha), Changsha, China; Neurology Department, Fujian Medical University Union Hospital, Fuzhou, China; Tuberculosis Department, The Affiliated Changsha Hospital of Xiangya School of Medicine, Central South University (The First Hospital of Changsha), Changsha, China; Rehabilitation Medicine Department, The Affiliated Changsha Hospital of Xiangya School of Medicine, Central South University (The First Hospital of Changsha), Changsha, China; Rehabilitation Medicine Department, The Affiliated Changsha Hospital of Xiangya School of Medicine, Central South University (The First Hospital of Changsha), Changsha, China

**Keywords:** cryptococcal meningitis, diagnosis, machine learning, nomogram, tuberculous meningitis

## Abstract

**Background:**

Differentiating cryptococcal meningitis from tuberculous meningitis is critical for initiating appropriate treatment and improving patient outcomes. However, overlapping pathogenic mechanisms, clinical presentations, laboratory findings, and imaging features complicate accurate diagnosis.

**Methods:**

We performed a retrospective single-center study at the First Hospital of Changsha, analyzing medical records from January 2021 to November 2024. A total of 271 patients were included: 125 diagnosed with cryptococcal meningitis and 146 with tuberculous meningitis. Using LASSO regression (least absolute shrinkage and selection operator), we identified 11 potential predictors, which were subsequently refined to 6 key variables: extracranial fungi, cerebrospinal fluid pressure, age, erythrocyte sedimentation rate, albumin levels, and India ink staining. A diagnostic nomogram was constructed through multivariate logistic regression and validated by receiver operating characteristic analysis, calibration plots, decision curve analysis, and SHAP tools (Shapley additive explanations) for model interpretability.

**Results:**

The optimized model incorporated 6 variables: extracranial fungal presence, cerebrospinal fluid pressure, patient age, erythrocyte sedimentation rate, albumin levels, and India ink staining. According to SHAP analysis, the significance of these factors in prediction was underscored. The model achieved an area under the curve of 0.919 for the training cohort and 0.921 for the validation cohort, demonstrating robust sensitivity and specificity across both data sets. Additionally, calibration plots and decision curve analysis validated the model's precision and its applicability in clinical settings.

**Conclusions:**

The developed nomogram accurately distinguishes cryptococcal meningitis from tuberculous meningitis with superior discriminative ability and clinical relevance, providing a valuable diagnostic tool for clinicians.

Cryptococcal meningitis (CM) and tuberculous meningitis (TBM) represent prevalent forms of meningitis encountered in clinical practice [1, 2]. Accurately distinguishing between these conditions is crucial for establishing effective treatment protocols and enhancing patient prognoses [3, 4]. However, the differentiation is complicated by significant overlaps in their pathogenic mechanisms, clinical symptoms, laboratory findings, and imaging characteristics [3, 5].

Pathogenically, CM is caused by the infiltration of *Cryptococcus* species into the central nervous system, predominantly affecting individuals with weakened immune systems, such as those with HIV or immunosuppressive treatments [6]. In contrast, TBM results from the hematogenous spread of *Mycobacterium tuberculosis* to the meninges, often originating from primary infection sites such as the lungs [7]. Clinically, CM and TBM present with nonspecific meningitic symptoms, such as headache, fever, vomiting, and neck stiffness, which can easily lead to diagnostic ambiguity. Laboratory evaluations show differences in cerebrospinal fluid (CSF) parameters—such as white blood cell (WBC) count, protein concentration, and glucose level—but these indicators can overlap in certain patients [8, 9], making it challenging to differentiate the 2 conditions solely based on these metrics. Furthermore, neuroimaging findings, including brain parenchymal lesions and meningeal enhancement, can be similar in both diseases [10], thereby reducing the diagnostic specificity of individual imaging techniques.

Although the cryptococcal latex agglutination test and lateral flow assay exhibit high sensitivity [11, 12], their widespread adoption in economically underdeveloped regions faces significant obstacles: prohibitive reagent costs, stringent cold chain transportation requirements, and the reliance on specialized laboratory infrastructure [13]. Therefore, diagnostic models based on routine clinical indicators can provide rapid and cost-effective decision-making aids for health care facilities in resource-limited settings. Previous studies have developed models for diagnosing CM or TBM individually [14, 15], but few have created models for differentiating between CM and TBM. This study aims to construct a diagnostic nomogram that integrates multifaceted clinical data to improve the accuracy of distinguishing patients with CM from TBM. The goal is to provide clinicians with a more reliable diagnostic tool, optimize patient diagnostic and treatment pathways, minimize the risks associated with misdiagnosis and inappropriate therapy, and ultimately enhance the quality of life for affected patients.

## MATERIALS AND METHODS

### Study Data

Ethical approval was granted by the Ethics Committee of the First Hospital of Changsha (approval LY-2023-029), which waived the requirement for informed consent due to the study's retrospective design. The research adhered strictly to the Helsinki Declaration principles, ensuring the protection of participants' rights, safety, and well-being throughout the investigation.

The investigation utilized hospital medical records and physician order systems to extract case data spanning January 2021 to November 2024, focusing on primary discharge diagnoses of CM and TBM. An initial pool of 336 patient files was assembled. After exclusion of 57 cases designated for inpatient review and 8 instances exhibiting CM and TBM, 271 records were included in the final analysis, comprising 125 patients diagnosed with CM and 146 with TBM.

### Study Design

The study involved creating a differential diagnosis model using the acquired data, assessing the model's efficacy, and analyzing its key variables. Compliance with the TRIPOD guidelines was maintained to ensure the robust development and validation of the predictive models [16]. The diagnostic criteria for CM encompassed several confirmation methods: the identification of *Cryptococcus* species in CSF through smear microscopy or India ink staining (IIS), isolation via CSF culture, and positive results from the cryptococcal latex agglutination assay [17]. Patients were included in the study if they were discharged with a primary diagnosis of CM or meningoencephalitis, substantiated by definitive etiologic evidence such as CSF capsular antigen testing, fungal culture, or CSF next-generation sequencing. Exclusion criteria consisted of individuals admitted solely for follow-up care, those with other intracranial infections, and patients who had not received antifungal therapy within 1 week prior to hospitalization.

For TBM, the diagnostic framework adhered to the 2010 South African expert consensus, utilizing a comprehensive scoring system that integrated clinical manifestations, CSF examination results, neuroimaging findings, evidence of extrapulmonary tuberculosis, and the exclusion of alternative diagnoses [18]. Regarding inclusion criteria, patients were included if they were discharged with TBM as the principal diagnosis, which adhered to the 2010 South African expert consensus, including definite, probable, and possible TBM cases (for diagnostic scoring criteria, see the supplementary materials). The exclusion criteria mirrored those for CM, excluding individuals admitted solely for follow-up, patients with alternative intracranial infections, and those who had not undergone antituberculosis treatment within 1 week prior to admission.

### Candidate Predictor Variables

Drawing on clinical expertise and established diagnostic criteria for intracranial infections [17, 19–21], we ensured the model's accuracy by collecting primary objective variables from patients upon admission. These variables encompass demographic data, blood biochemical markers, and CSF analysis parameters. Specifically, demographic information includes age, sex, and the presence of vomiting at disease onset, as well as histories of diabetes, HIV, tumors, extrapulmonary tuberculosis, and fungal infections. The blood biochemical markers gathered consist of WBC, red blood cell, and platelet counts; hemoglobin levels; neutrophil, lymphocyte, and monocyte count and percentages; and procalcitonin, C-reactive protein, erythrocyte sedimentation rate (ESR), and albumin levels. Additionally, the CSF analysis involves measuring CSF pressure, WBC count, proportions of polymorphonuclear cells and monocytes, protein concentration, glucose level, and chloride concentration, as well as IIS and acid-fast staining. These clinical parameters are routinely employed in the diagnosis and management of diseases, and their readily accessible nature supports the clinical implementation and promotion of the developed model.

### Machine Learning

To accurately identify key predictors associated with CM, we implemented the LASSO regression technique (least absolute shrinkage and selection operator), which combines variable selection and regularization. This method effectively navigates the challenges of multicollinearity among variables, enhancing the stability and predictive accuracy of the model. Following this, a 10-fold cross-validation was carried out to determine the optimal tuning parameter (λ) within the LASSO framework. Through a rigorous algorithm, the most influential predictors were carefully selected. The data set was subsequently split into training and testing subsets in a 6:4 ratio. Within the training subset, the LASSO-selected predictors were integrated into a multivariate logistic regression model, and a nomogram was developed to illustrate the impact of these predictors. To streamline the model, predictors with minimal effects were removed, and the remaining variables underwent another round of multivariate logistic regression, followed by the construction of an updated nomogram.

### Decision Curve Analysis

Moreover, the prediction model was evaluated for its discriminatory capacity, precision, and clinical relevance within training and testing cohorts. The model's ability to differentiate was quantified by an area under the receiver operating characteristic curve (AUC). Calibration plots were generated to assess the alignment between anticipated probabilities and actual outcomes. Subsequently, decision curve analysis was performed to determine the model's clinical applicability. Additionally, the model's predictors were elucidated, and SHAP values (Shapley additive explanations) were calculated for each variable. The predictive performance of these factors was visually depicted through global importance, swarm, waterfall, and force plots, thereby enhancing the comprehensive understanding of the model.

### Statistical Analysis

In this study, we performed statistical analyses and created figures using R software version 4.4.2, developed by Ross Ihaka and Robert Gentleman at the University of Auckland, New Zealand. The data set was evaluated by median (IQR) and mean ± SD as descriptive statistics. For binary variables (eg, HIV status, diabetes, extracranial tuberculosis), a Pearson chi-square test was used. When expected cell counts were <5 in any contingency table cell (eg, tumor: n = 1 in TM group), a Fisher exact test was applied instead. For nonnormally distributed continuous variables (eg, age, WBC, CSF pressure), a Mann-Whitney *U* test (Wilcoxon rank sum test) was used and reported as median (IQR). Normally distributed variables were analyzed with a Student *t* test (eg, red blood cells, hemoglobin) and reported as mean ± SD. *P* < .05 was considered to indicate statistical significance.

## RESULTS

### Clinical Characteristics of Patients With TBM

**Figure 1.**
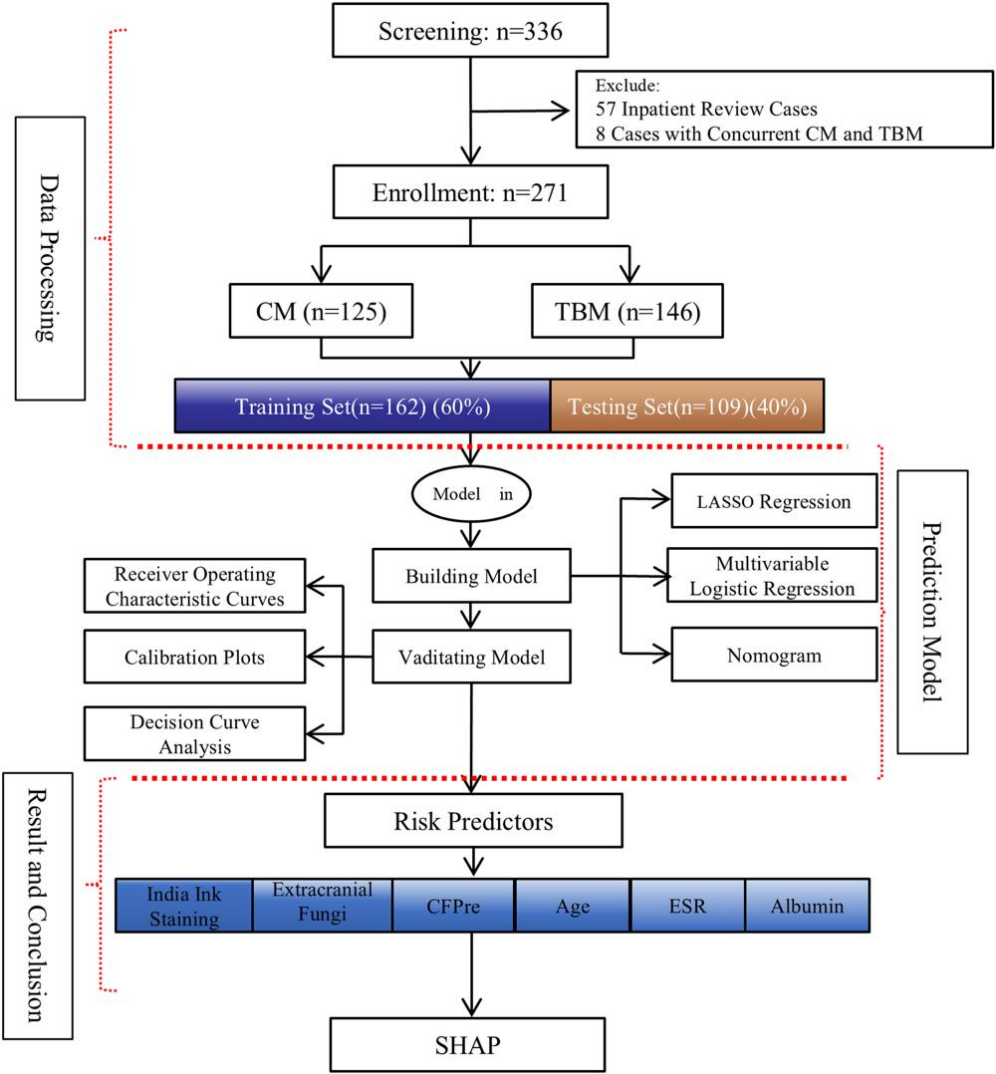
Flow diagram of participant inclusion and exclusion criteria. A total of 271 patients with meningitis were screened: 146 were diagnosed with tuberculous meningitis (TBM) and 125 with cryptococcal meningitis (CM) following laboratory and clinical confirmation. Abbreviations: CFPre, cerebrospinal fluid pressure; ESR, erythrocyte sedimentation rate; LASSO, least absolute shrinkage and selection operator; SHAP, Shapley additive explanations.

The study's workflow is illustrated in Figure 1. An overall 271 case records were included in the analysis, as detailed in Table 1. Among these, 125 individuals were diagnosed with CM and 146 with TBM. The CM and TBM cohorts exhibited a significantly higher male predominance as compared with females. The mean age across the entire population was 43 years, with the CM group being younger on average than the TBM group. People with HIV were a majority in both cohorts. No significant differences were observed between the CM and TBM groups regarding sex distribution or prior diagnosis of diabetes or tumor; red blood cell or platelet count; hemoglobin, C-reactive protein, procalcitonin, or albumin level; or neutrophil, monocyte, or lymphocyte percentage. Additionally, parameters such as the proportion of monocytes in CSF, chloride concentration in CSF, and acid-fast staining did not differ significantly between the groups. However, notable distinctions were found between the CM and TBM populations in variables such as age, HIV status, presence of extrapulmonary tuberculosis, fungal infections, occurrence of vomiting at disease onset, WBC count, ESR, and IIS; percentage of neutrophils, lymphocytes, and monocytes; and CSF analysis in terms of pressure, WBC count, proportion of polymorphonuclear cells, glucose level, protein concentration, and chloride concentration.

**Table 1.** Initial Clinical Characteristics of Patients With Cryptococcal and Tuberculous Meningitis at Admission

Variable	Total (n = 271)	TBM (n = 146)	CM (n = 125)	*P* Value
Sex				.46
Female	45 (17)	27 (18)	18 (14)	
Male	226 (83)	119 (82)	107 (86)	
HIV				<.001
No	64 (24)	51 (35)	13 (10)	
Yes	207 (76)	95 (65)	112 (90)	
Diabetes				>.99
No	262 (97)	141 (97)	121 (97)	
Yes	9 (3)	5 (3)	4 (3)	
Extracranial tuberculosis				<.001
No	140 (52)	53 (36)	87 (70)	
Yes	131 (48)	93 (64)	38 (30)	
Tumor				>.99
No	270 (100)	145 (99)	125 (100)	
Yes	1 (0)	1 (1)	0 (0)	
Extracranial fungi				.003
No	232 (86)	134 (92)	98 (78)	
Yes	39 (14)	12 (8)	27 (22)	
Vomit				.003
No	225 (83)	131 (90)	94 (75)	
Yes	46 (17)	15 (10)	31 (25)	
India ink staining				<.001
Positive	202 (75)	146 (100)	56 (45)	
Negative	69 (25)	0 (0)	69 (55)	
Acid-fast staining				>.99
Negative	271 (100)	146 (100)	125 (100)	
Age, y	43 (32–56)	46 (32.25–60)	41 (30–50)	.007
WBC, 10^9^/L	4.95 (3.5–6.69)	5.46 (4.3–6.92)	4.6 (3.02–6.17)	.001
RBC, 10^12^/L	3.65 ± 0.79	3.65 ± 0.84	3.65 ± 0.72	.979
Platelet, 10^9^/L	186 (127–242)	184 (130.5–238.5)	189 (124–243)	.684
Neutrophil				
Percentage	71.9 (60–81.85)	74 (59.52–83.25)	71.5 (61.1–79.6)	.357
Count, 10^9^/L	3.55 (2.08–5.08)	3.87 (2.42–5.5)	3.02 (1.83–4.58)	.004
Monocyte				
Percentage	7.8 (5.55–10.55)	7.55 (5.53–10.23)	8.2 (5.6–10.7)	.505
Count, 10^9^/L	0.38 (0.27–0.53)	0.4 (0.3–0.57)	0.36 (0.25–0.49)	.018
Lymphocyte				
Percentage	16.1 (9.85–27.35)	15.65 (9.22–26.9)	16.5 (10–27.8)	.665
Count, 10^9^/L	0.79 (0.5–1.21)	0.86 (0.55–1.46)	0.69 (0.45–1.12)	.023
Hemoglobin, g/L	110.31 ± 24.83	111.47 ± 26.78	108.94 ± 22.36	.398
CRP, mg/L	11.9 (4.65–33.44)	11.39 (4.32–38.45)	12 (5.1–26.26)	.87
PCT, ng/mL	0 (0–0.14)	0 (0–0.21)	0 (0–0.11)	.269
ESR, mm/h	66 (20–105)	46 (11–99)	85 (40–110)	<.001
Albumin, g/L	35.4 (30.7–39.35)	35.35 (30.45–39.18)	35.6 (30.9–39.5)	.306
CSF				
Pressure, mm H_2_O	170 (120–270)	150 (110–190)	250 (160–360)	<.001
WBC, 10^6^/L	15 (8–36.5)	18 (10–39.75)	12 (6–33)	.048
Proportion of cells				
Polymorphonuclear	20 (10–30)	20.5 (13–30)	20 (10–25)	.006
Mononuclear	80 (70–89)	79 (70–87)	80 (70–90)	.105
Protein, mg/L	846.4 (440.45–1377.9)	1007.05 (550.82–1642.67)	592 (331.9–1021)	<.001
Glucose, mmol/L	2.76 (2.1–3.29)	2.9 (2.3–3.4)	2.54 (2–3.11)	.01
Chloride, mmol/L	121.1 (116.1–124.1)	121.15 (115.53–124.47)	121 (117.7–123.9)	.522

Data are presented as No. (%), median (IQR), or mean ± SD.

Abbreviations: CM, cryptococcal meningitis; CRP, C-reactive protein; CSF, cerebrospinal fluid; ESR, erythrocyte sedimentation rate; PCT, procalcitonin; RBC, red blood cell; TBM, tuberculous meningitis; WBC, white blood cell.

### Development of Prediction Model

**Figure 2.**
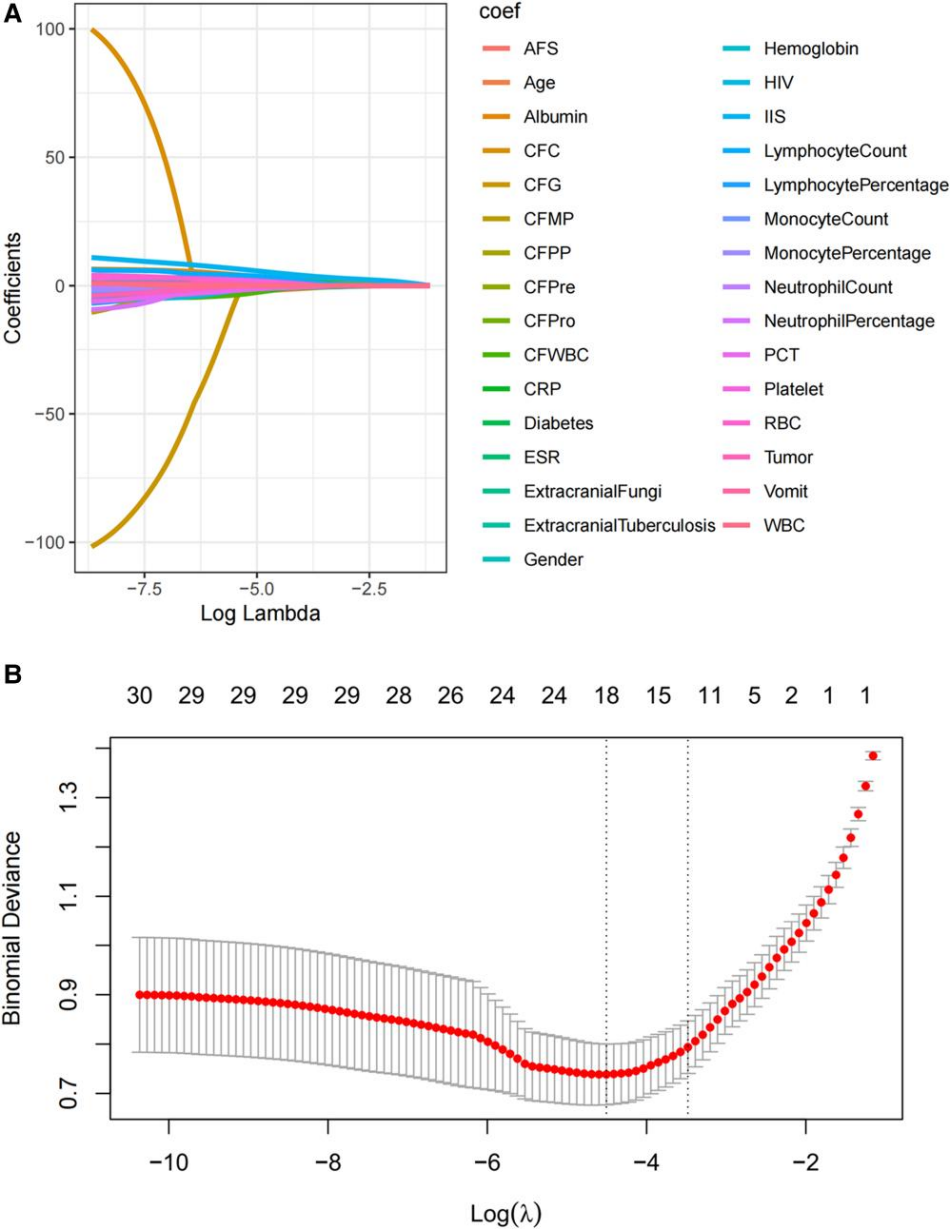
Machine learning. *A*, LASSO coefficient path plot: coefficient shrinkage paths for LASSO regression. *B*, Cross-validation curve for LASSO regression analysis: mean squared error vs log(λ). The vertical dashed line represents the optimal lambda (λ) value selected via 10-fold cross-validation, balancing model complexity and diagnostic accuracy. The optimal λ minimizes the mean squared error while avoiding overfitting. Error bars indicate SD across cross-validation folds. Features with nonzero coefficients at optimal λ were retained as predictors. Abbreviations: AFS, acid-fast staining; CFC, cerebrospinal fluid chloride; CFG, cerebrospinal fluid glucose; CFMP, cerebrospinal fluid monocytes; CFPP, cerebrospinal fluid polymorphonuclear cells; CFPre, cerebrospinal fluid pressure; CFPro, cerebrospinal fluid protein; CFWBC, cerebrospinal fluid white blood cells; CRP, C-reactive protein; ESR, erythrocyte sedimentation rate; IIS, India ink staining; LASSO, least absolute shrinkage and selection operator; PCT, procalcitonin; RBC, red blood cells; WBC, white blood cells.

**Figure 3.**
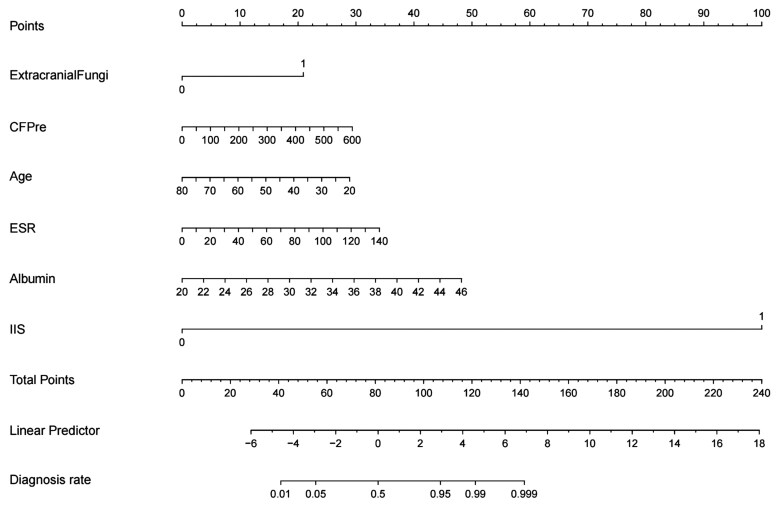
Nomogram for differentiating cryptococcal meningitis (CM) from tuberculous meningitis. Clinicians can assign points for each variable (eg, cerebrospinal fluid pressure, HIV status) and sum them to predict the probability of CM. Higher total points indicate increased likelihood of CM. Abbreviations: CFPre, cerebrospinal fluid pressure; ESR, erythrocyte sedimentation rate; IIS, India ink staining.

Cross-validation analysis revealed that with an increase in the regularization parameter lambda (λ), the model error initially declined and subsequently rose (Figure 2*A*). We chose a λ value set to λ_min + 1 SE, which resulted in a more parsimonious and refined model with fewer variables. Regression coefficients were determined by utilizing λ_min + 1 SE, leading to the identification of 11 variables with nonzero coefficients as diagnostic predictors for CM (Figure 2*B*): HIV status, extracranial fungal infection, neutrophil percentage, CSF protein concentration, CSF pressure, extracranial tuberculosis, age, ESR, albumin levels, lymphocyte count, and IIS. These 11 predictors were then incorporated into a multivariate regression model, and a nomogram was developed. The nomogram illustrated the predictive performance of each predictor, facilitating the removal of those with limited predictive value. Ultimately, 6 predictors were retained: extracranial fungi, CSF pressure, age, ESR, albumin, and IIS (Figure 3). These 6 variables were reintroduced into a multivariate regression analysis to construct the final nomogram (Table 2).

**Table 2.** Multivariable Logistic Regression Analysis of Predictors for Cryptococcal Meningitis

Variable	Coefficient^aa^	SE	Wald *Z*	Pr(>|*Z*|)^b^
Intercept	−9.0882	2.67	−3.4	.0007
Extracranial fungi = 1^c^	2.3849	0.8424	2.83	.0046
CSF pressure, mm H_2_O	0.0056	0.0024	2.31	.0207
Age	−0.0549	0.0199	−2.75	.0059
ESR, mm/h	0.0277	0.0073	3.82	.0001
Albumin, g/L	0.2114	0.0614	3.44	.0006
India ink staining = 1^c^	11.4047	22.4639	0.51	.6117

Results from a multivariable logistic regression model analyzing associations between clinical variables and cryptococcal meningitis.

Abbreviations: CSF, cerebrospinal fluid; ESR, erythrocyte sedimentation rate.

^a^Coefficients are expressed in log-odds units; a positive value indicates increased odds of the outcome.

^b^Pr(>|*Z*|): *P* values derived from Wald tests for individual coefficients.

^c^Categorical variables (extracranial fungi and India ink staining) were coded as 1 = present and 0 = absent.

### Identification and Validation of Key Diagnostic Predictors for Cryptococcal Meningitis

**Figure 4.**
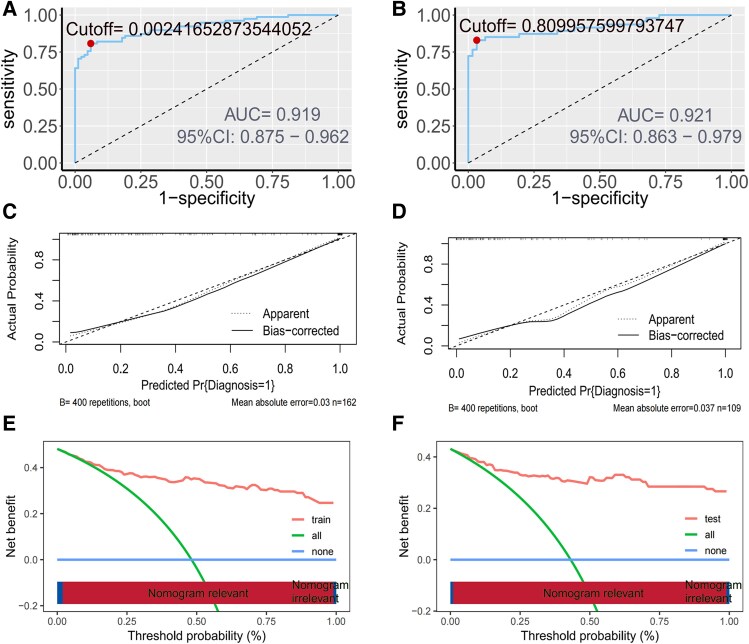
*A* and *B*, AUCs for the diagnostic model in training and testing sets, respectively. The AUC quantifies discrimination ability: training, 0.919; testing, 0.921. *C* and *D*, Calibration curves assess agreement between predicted probabilities and observed outcomes in the training and testing cohorts, respectively. Diagonal dashed line represents random chance. Long-dashed line represents perfect calibration. *E* and *F*, DCA curves show the net clinical benefit of the diagnostic model across threshold probabilities in the training and testing cohorts, respectively. The green line represents “treat all as cryptococcal meningitis” and the blue line “treat none.” Abbreviations: AUC, area under the receiver operating characteristic curve; DCA, decision curve analysis.

The discriminatory capability of the predictive model was assessed by calculating AUC values. As depicted in Figure 4*A* and 4*B*, the model achieved an AUC of 0.919 within the training cohort, demonstrating a sensitivity of 80.8% and a specificity of 94.0%. In the validation cohort, the AUC was 0.921, with sensitivity and specificity values of 83.0% and 96.8%, respectively. Calibration analysis revealed strong agreement between the predicted and observed probabilities of CM across the training and testing data sets. Specifically, the training set exhibited a mean absolute error of 0.03, a mean squared error of 0.0013, and a 90th-percentile absolute error of 0.059 (Figure 4*C* and 4*D*). Similarly, the testing set showed a mean absolute error of 0.037, a mean squared error of 0.00226, and a 90th-percentile absolute error of 0.079. The clinical utility of the model was further evaluated in decision curve analysis (Figure 4*E*), which demonstrated that the prediction model yielded significantly higher net benefits when compared with the 2 extreme scenarios in the internal validation set (Figure 4*F*). These outcomes indicate that the nomogram provides enhanced net benefits and superior accuracy in prediction.

### Analysis of Key Feature Variables With SHAP Values and Individual Case Contribution

**Figure 5.**
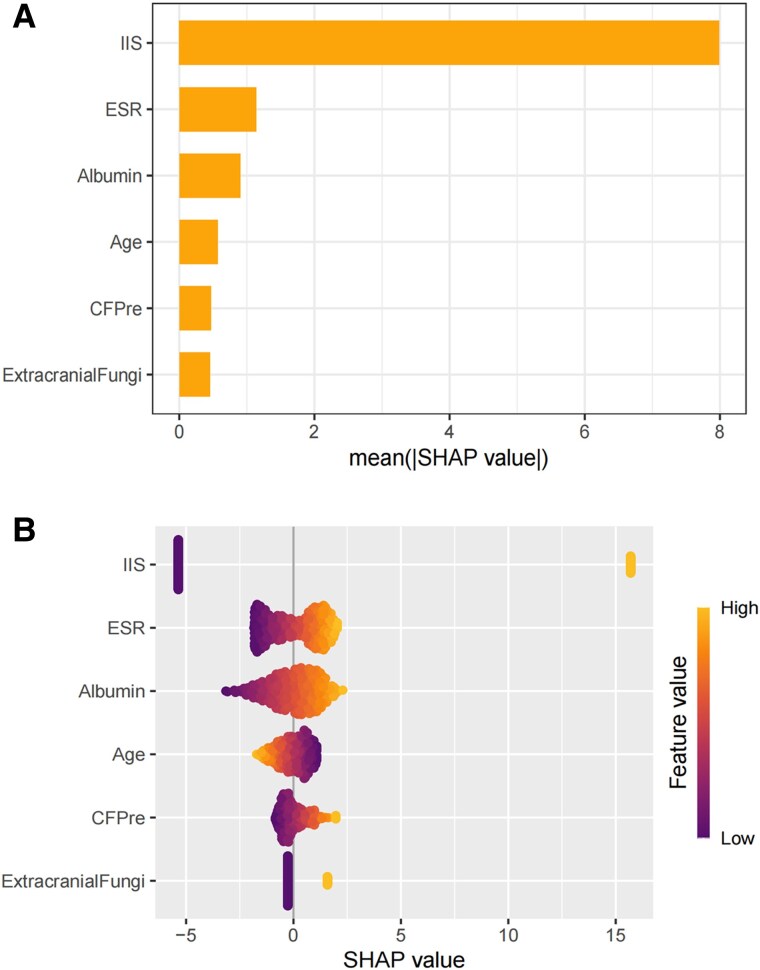
*A*, Global importance plot: SHAP summary plot ranking the importance of features in the model. Higher mean SHAP values indicate stronger influence on predictions. Positive SHAP values drive predictions toward cryptococcal meningitis and negative toward tuberculous meningitis. *B*, Swarm plot: distribution of SHAP values per feature across all samples. Overlapping dots are jittered for visibility. Abbreviations: CFPre, cerebrospinal fluid pressure; ESR, erythrocyte sedimentation rate; IIS, India ink staining; SHAP, Shapley additive explanations.

**Figure 6.**
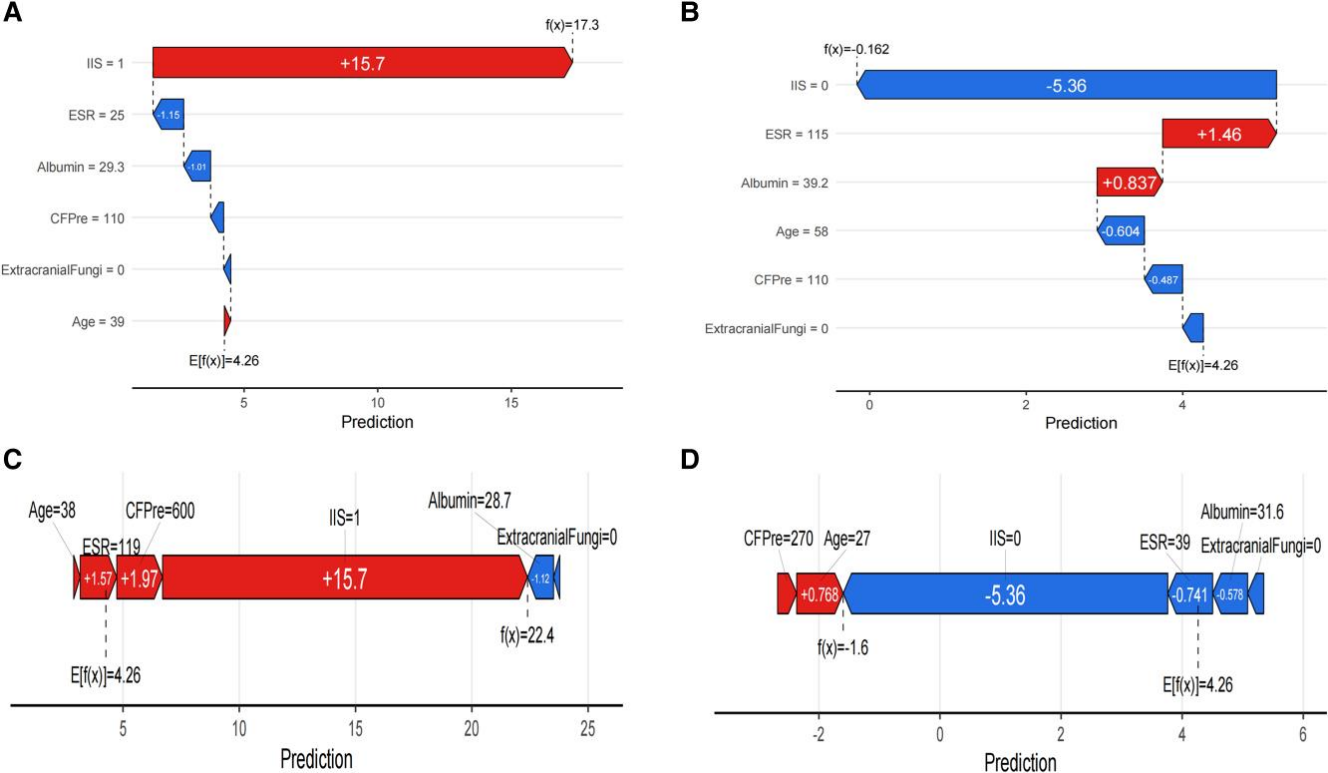
*A* and *B*, Waterfall plots illustrate how each feature contributes to shifting the baseline prediction (population average) to the final prediction for 2 representative patients. Red bars increase cryptococcal meningitis probability; blue bars decrease it. *C* and *D*, Force plots depict feature contributions for the same patients. Arrows indicate the direction and magnitude of influence for each feature, with the final prediction probability displayed on the right. Abbreviations: CFPre, cerebrospinal fluid pressure; ESR, erythrocyte sedimentation rate; IIS, India ink staining.

The model's 6 key feature variables were analyzed by SHAP value calculations. Through the application of global importance and swarm plots (Figure 5*A*), we validated the overall predictive power of these variables across the entire data set. Specifically, extracranial fungi, CSF pressure, ESR, albumin, and IIS demonstrated a positive influence on the model's predictions, whereas age showed a negative impact (Figure 5*B*). To further illustrate the contribution of each predictor on an individual level, we randomly selected 4 cases and employed waterfall and force plots (Figure 6*A* and 6*B*), thereby highlighting the role of the 6 features in each selected sample (Figure 6*C* and 6*D*).

## DISCUSSION

This research successfully established and validated a diagnostic nomogram to differentiate CM and TBM, marking a significant advancement in clinical diagnostics. Unlike previous studies that primarily focused on unidimensional indicators such as solely CSF biochemical markers or clinical symptoms [22–24], our model uniquely incorporates a comprehensive set of multidimensional variables, such as demographic factors (eg, age, sex) and medical histories (including HIV status, malignancies, tuberculosis), alongside blood and CSF biochemical analyses. This integrative approach captures the intricate disease characteristics more effectively, thereby improving diagnostic accuracy. For example, HIV infection status shows a marked difference between patients with CM and TBM, aligning with existing literature [25–27]. Given that individuals who are immunocompromised are more prone to CM [28], this factor provides essential discriminative power, thereby enhancing the model's diagnostic performance.

The developed model showcases outstanding efficacy across diverse metrics. The AUC achieves 0.919 for the training cohort and 0.921 for the validation cohort, indicating a strong ability to accurately distinguish between CM and TBM. Calibration analysis demonstrates a high level of agreement between the forecasted probabilities and the observed outcomes, reflecting superior predictive accuracy. Using this model, health care professionals can reliably assess the likelihood of disease presence in patients, thereby enhancing diagnostic decision making. For instance, a high predicted probability can prompt the immediate initiation of targeted therapies, preventing unnecessary delays. The model's clinical robustness, validated through decision curve analysis, presents a significant net benefit as compared with baseline scenarios. This enables clinicians to weigh the pros and cons of various diagnostic strategies, make informed choices regarding diagnostic and treatment plans, and improve the efficiency of medical resource utilization alongside patient treatment outcomes.

The analysis of model variables holds significant clinical importance. SHAP value analysis facilitates the determination of each variable's individual impact and direction. IIS positivity serves as the most effective predictor, as it is a fundamental diagnostic criterion for CM [17]. Nonetheless, our research identified a mere 55% positivity rate for IIS during the initial lumbar puncture, leading to a considerable number of false negatives [29]. Extracranial fungal infection is a strong predictor: a positive outcome greatly indicates a high probability of CM [30] because fungal colonization is closely linked to the mechanism of fungal invasion, increasing the risk of infection and providing substantial diagnostic evidence. CSF pressure is also critical [31]. In patients with CM, intracranial pressure often rises due to obstructions in CSF flow, and irregular pressure variations help differentiate disease types and guide clinical monitoring and treatment adjustments [32]. The ESR reflects inflammatory responses, potentially indicating a more severe inflammatory reaction in CM as opposed to TBM, although further studies are needed for validation [33, 34]. Albumin levels represent nutritional status, potentially suggesting that *M tuberculosis* causes more significant bodily depletion than *Cryptococcus* [35, 36]. Age has a negative impact. While HIV was identified as a predictor for CM, it was excluded due to its relatively low predictive effectiveness. This exclusion may result from the higher prevalence of HIV among patients with CM and the younger age demographic among the people with HIV [37], thereby contributing to the observed negative effect of age. Expanding the sample size in future studies could enhance the comprehensive understanding of age's predictive strength regarding CM.

However, this research possesses specific limitations. The data set stems from a retrospective analysis conducted at a single institution, restricting the sample's generalizability due to factors such as geographic location, varying levels of hospital diagnostics and treatments, and potential biases in patient selection. Expanding to multicenter studies with larger cohorts could enhance the study's applicability. Additionally, some variables contain missing data. Although these gaps have been addressed, they may still affect the model's precision and stability. Refining data collection and processing techniques could reduce these impacts. The current model is based on established clinical indicators and does not include emerging biomarkers, such as particular gene expressions and metabolite profiles. Integrating these biomarkers may further improve diagnostic accuracy and so represents a direction for future research.

Overall, this model serves as an effective tool for diagnosing CM and TBM, aiding clinicians in making accurate decisions and advancing the field of precision medicine for meningitis. Future studies should aim to optimize the model, collaborate across multiple centers to build extensive databases, incorporate innovative biomarkers, elevate diagnostic standards, enhance patient outcomes, and contribute to the global efforts in preventing and treating meningitis.

## CONCLUSION

This investigation has effectively created and substantiated a nomogram for differentiating CM from TBM. By amalgamating key demographic and clinical factors, the model demonstrates superior accuracy and robust calibration. Decision curve analysis confirms its advantageous clinical utility. The application of SHAP values alongside visual tools enhances the interpretability of the model, providing clinicians with a reliable differential diagnostic framework and significantly advancing the application of precision medicine in the management of meningitis.

## Supplementary Material

ofaf217_Supplementary_Data

## Supplementary Data

Supplementary materials are available at *Open Forum Infectious Diseases* online. The original data in the Supplementary material are from the clinical data of the First Hospital of Changsha. Consisting of data provided by the authors to benefit the reader, the posted materials are not copyedited and are the sole responsibility of the authors, so questions or comments should be addressed to the corresponding author.

## Notes

***Acknowledgments.*** We extend our profound appreciation to all study participants and the dedicated staff at the First Hospital of Changsha for their work in data acquisition and management.

***Author contributions*.** F. L.: methodology, software, visualization, data curation, formal analysis, funding acquisition, writing–original draft. C. C.: investigation, resources, funding acquisition. Y. L.: Writing–review and editing. H. L.: conceptualization, methodology, supervision, validation, writing–review and editing.

***Data availability***. The authors confirm that the data supporting the findings of this study are available within the supplementary materials. For inquiries regarding data access or requests to obtain the data, please contact the corresponding author: Hua Liu at lhcssdyy@126.com.

***Ethics approval and consent to participate*.** Ethical approval was granted by the Ethics Committee of the First Hospital of Changsha (approval LY-2023-029). Due to the retrospective nature of the study, the Ethics Committee of the First Hospital of Changsha waived the need for obtaining informed consent. This research adheres strictly to the Helsinki Declaration guidelines, ensuring the protection of participants’ rights, safety, and well-being throughout the study.

***Financial support*.** This work was supported by the Hunan Provincial Natural Science Foundation of China (grant 2024JJ9510); and the Health Commission of Changsha (No. KJ-B2023043).
